# Fluxomics of the Eastern Oyster for Environmental Stress Studies

**DOI:** 10.3390/metabo4010053

**Published:** 2014-01-07

**Authors:** Andrey P. Tikunov, Michael K. Stoskopf, Jeffrey M. Macdonald

**Affiliations:** 1 Joint Department of Biomedical Engineering, NC State University and UNC Chapel Hill, Chapel Hill, NC 27599, USA; E-Mails: michael_stoskopf@ncsu.edu (M.K.S.); jeffrey_macdonald@med.unc.edu (J.M.M.); 2 Environmental Medicine Consortium, NC State University, 4700 Hillsborough Street, Raleigh, NC 27606, USA; 3 Department of Clinical Sciences, College of Veterinary Medicine, North Carolina State University, 4700 Hillsborough Street, Raleigh, NC 27606, USA

**Keywords:** ^1^H & ^13^C NMR, metabolomic, fluxomic, oyster, mass balance, 2-^13^C/^15^N-glycine, U-^13^C-glucose

## Abstract

The metabolism of 2-^13^C/^15^N-glycine and U-^13^C-glucose was determined in four tissue blocks (adductor muscle, stomach and digestive gland, mantle, and gills) of the Eastern oyster (*Crassostrea virginica*) using proton (^1^H) and carbon-13 (^13^C) nuclear magnetic resonance (NMR) spectroscopy. The oysters were treated in aerated seawater with three treatments (5.5 mM U-^13^C-glucose, 2.7 mM 2-^13^C/^15^N-glycine, and 5.5 mM U-^13^C-glucose plus 2.7 mM 2-^13^C/^15^N-glycine) and the relative mass balance and ^13^C fractional enrichments were determined in the four tissue blocks. In all tissues, glycine was metabolized by the glycine cycle forming serine exclusively in the mitochondria by the glycine cleavage system forming 2,3-^13^C-serine. In muscle, a minor amount of serine-derived pyruvate entered the Krebs cycle as substantiated by detection of a trace of 2,3-^13^C-aspartate. In all tissues, U-^13^C-glucose formed glycogen by glycogen synthesis, alanine by glycolysis, and glutamate and aspartate through the Krebs cycle. Alanine was formed exclusively from glucose via alanine transaminase and not glycine via alanine-glyoxylate transaminase. Based on isotopomer analysis, pyruvate carboxylase and pyruvate dehydrogenase appeared to be equal points for pyruvate entry into the Krebs cycle. In the 5.5 mM U-^13^C-glucose plus 2.7 mM 2-^13^C/^15^N-glycine emergence treatment used to simulate 12 h of “low tide”, oysters accumulated more ^13^C-labeled metabolites, including both anaerobic glycolytic and aerobic Krebs cycle intermediates. The aerobic metabolites could be the biochemical result of the gaping behavior of mollusks during emergence. The change in tissue distribution and mass balance of ^13^C-labeled nutrients (U-^13^C-glucose and 2-^13^C/^15^N-glycine) provides the basis for a new quantitative fluxomic method for elucidating sub-lethal environmental effects in marine organisms called whole body mass balance phenotyping (WoMBaP*).*

## 1. Introduction

Our understanding of organ specific toxicity is primarily based on a combination of traditional toxicokinetic studies that follow absorption, distribution, biotransformation and excretion of xenobiotics and toxicodynamic studies. The central concept is that perturbed metabolic pathways will result in detectable changes in the mass balance of metabolites compared to baseline conditions. From this concept comes the corollary that careful comprehensive quantification of these perturbations can elucidate the biochemical basis of the toxicity. Typically ^14^C tracers have been used in environmental toxicology to study comprehensive distribution and disposition of xenobiotics in whole organisms [1,2]. More recently, highly sensitive analytical platforms including NMR spectroscopy and mass spectrometry have allowed using stable isotopes for metabolic pathway analysis. When physiological concentrations of metabolites are within the range of ^13^C NMR spectroscopic sensitivity, real-time, noninvasive, *in situ*, metabolic distribution and disposition studies [3] can generate comprehensive toxicodynamic data. In these situations ^13^C NMR spectroscopy of ^13^C labeled nutrients can be used to determine the metabolic effects of environmental stressors including toxicants. These techniques make it possible to use a single time point analysis to study time-dependent changes in the wide array of biochemical reaction velocities of an organism’s metabolism, or the fluxome [4]. This is the basis of whole body mass balance phenotyping (WoMBaP), a new toxicodynamic method that quantitatively expresses the character of the metabolome and the fluxome as they respond to an environmental stress. A previous metabolomic study using ^1^H NMR spectroscopy has identified the biochemical profile of the Eastern oyster (*Crassostrea virginica*) [5]. This study [5] provides the basis for developing WoMBaP for this species, potentially including non-invasive, *in vivo* WoMBaP studies that would use localized NMR spectroscopy [6,7] or chemical shift imaging (CSI) of intact oysters [3], to determine the metabolic contribution of integrated functional organ systems. This ability to sample living oysters would permit longitudinal *in vivo* NMR or CSI studies of chemical dynamics to facilitate the interpretation of the biochemical impacts of target organ disease or intoxication [8,9,10,11].

This is the first comprehensive ^13^C fluxomic study of oysters using two metabolically important ^13^C-labeled nutrients, glucose and glycine. Therefore, the major goal of this study was to establish baseline ^13^C distributions derived from U-^13^C-glucose and 2-^13^C/^15^N-glycine among oyster tissues, and to demonstrate the feasibility of using WoMBaP in the oyster model. Specifically, Eastern oysters were exposed to three different treatments (2-^13^C/^15^N-glycine, U-^13^C-glucose, and ^13^C/^15^N-glycine + U-^13^C-glucose) and four dissected organ blocks were analyzed by ^13^C/^1^H-NMR-based WoMBaP: (1) adductor muscle; (2) mantle; (3) gills; and (4) the gastrointestinal (GI) tract including the stomach and digestive gland. An initial ^1^H NMR metabolomic study demonstrated that the osmolyte betaine was the most abundant metabolite in all tissues, as were primary metabolites of the Krebs cycle and the end product of glycolysis, which is alanine rather than lactate in oyster [5]. We chose glucose to probe glycolysis and the Krebs cycle, while glycine was chosen to probe betaine, which is trimethylglycine, as well glutathione metabolism because glutathione has been the focus of many toxicology studies with oysters [12,13,14]. In addition, glycine forms serine, and a recent publication has been shown that the ^13^C serine isotopomers formed from 2-^13^C-glycine can be used to determine the percent produced in the mitochondria *versus* cytosol, as well as the mitochondrial redox status [15]. Finally, a recent comprehensive transcriptomics publication on the Pacific oyster (*C. gigas*) suggested that a primary pathway for alanine synthesis is via the alanine-glyoxylate transamination (AGT) pathway [16], wherein the ^15^NH_3_ from 2-^13^C/^15^N-glycine will combine with pyruvate forming alanine and glyoxylate. Therefore, the relative contribution of alanine derived from AGT and alanine amino transaminase (ALT) will be easily detected by ^13^C NMR analysis of oysters fed 2-^13^C/^15^N-glycine and U-^13^C-glucose.

## 2. Results and Discussion

### 2.1. Comparison of the Perchloric Acid versus Methanol Extraction Methods Applied to Oyster Tissues

**Figure 1:**
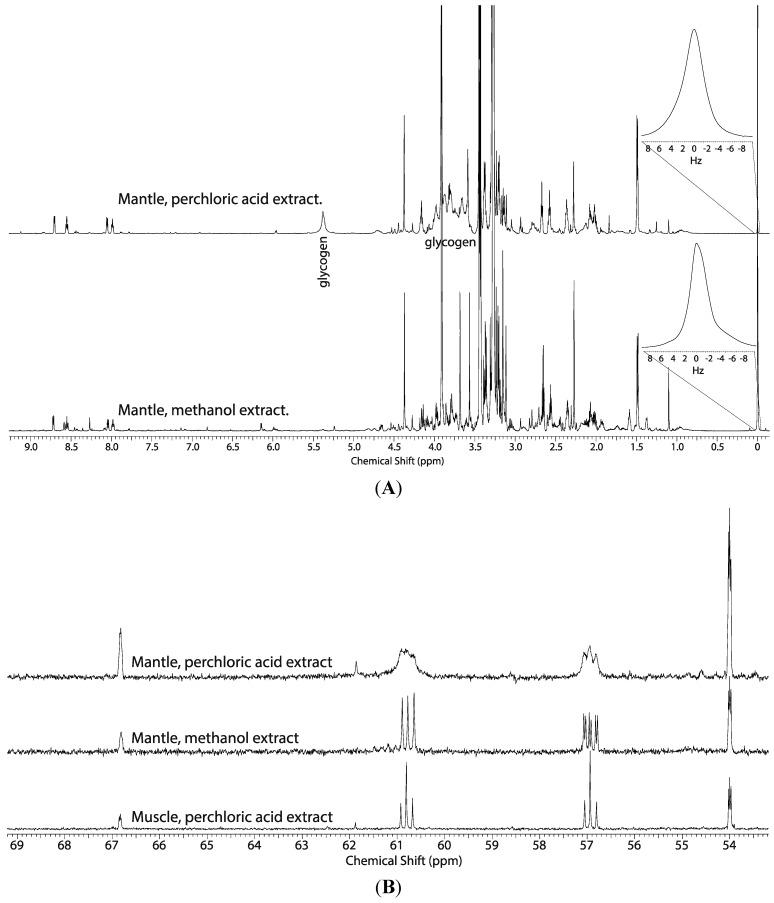
(**A**) Representative ^1^H NMR spectra of the mantle tissue block of oysters exposed to U-^13^C-glucose and extracted with 8% perchloric acid showing the broad resonances between 5.42 ppm and 3.25 ppm (top) and the increase in resolution in the same region due to precipitation of glycogen during extraction with methanol (bottom). (**B**) Representative portions of the ^13^C NMR spectra of the mantle tissue block from oysters exposed to seawater containing 2.7 mM 2-^13^C/^15^N-glycine and extracted with perchloric acid (top) and 50% methanol (middle), and perchloric acid extract of muscle (bottom) from oysters exposed to seawater containing 2.7 mM 2-^13^C-glycine.

**Figure 2:**
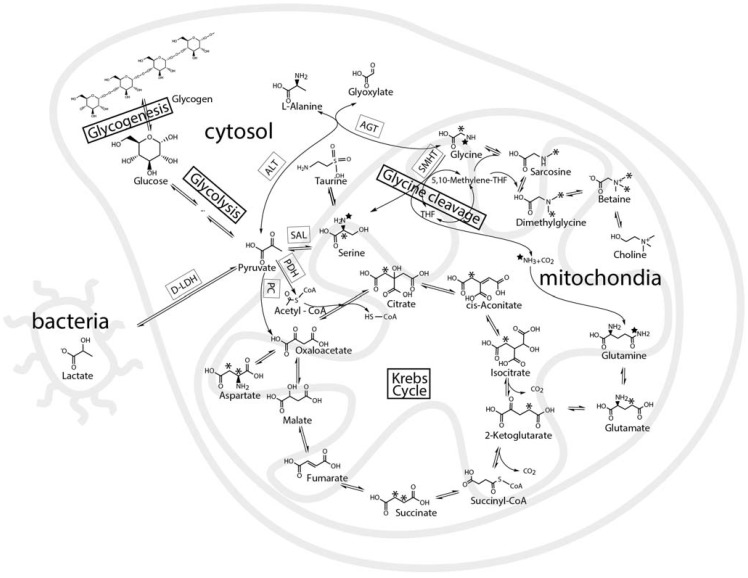
A schematic biochemical representation of the various known metabolic pathways in the oyster biosystem that were expected to be probed by U-^13^C-glucose and 2-^13^C/^15^N-glycine. The schematic map shows glycolysis, Krebs cycle, glycine and serine metabolism expected in the cell cytosol, mitochondria or symbiotic bacteria. The star and asterisk track the position of the ^13^C and ^15^N in metabolites of 2-^13^C/^15^N-glycine. The ^13^C-labeled metabolites of U-^13^C-glucose are not shown, but it would uniformly label glycogen, alanine, while the labeling pattern of glutamate formed by the Krebs cycle is discussed in the text.

Abbreviations: AGT, alanine-glyoxylate transferase (EC 2.6.1.44); ALT, Alanine transaminase (EC 2.6.1.2); Glycine cleavage system [glycine dehydrogenase (GLDC) EC 1.4.4.2; aminomethyltransferase (AMT) EC 2.1.2.10]; SHMT, serine hydroxymethyl transferase (EC 2.1.2.1); L-serine ammonia lyase (SAL, EC 4.3.1.17); D-LDH, D-lactate dehydrogenase; PC, pyruvate carboxylase; PDH, pyruvate dehydrogenase.

Previous ^1^H NMR metabolomic studies have revealed that mantle and the digestive gland/stomach tissue blocks contain a large amount of glycogen (0.5–7 mg of glycogen per 100 g of oyster tissue, depending on the season), but adductor muscle does not [5]. Recent extraction studies have shown that perchloric acid extraction of glycogen is efficient [4]. Glycogen confounds spectral analysis by co-resonating with other metabolites between 3.25–5.42 ppm, such as serine and glycine. Perchloric acid extraction was used in the initial studies (Figure 1A—top spectrum), but to simplify the ^1^H NMR spectra and more accurately quantify ^13^C fractional enrichments of glycine, betaine, glutathione, serine, and potentially other metabolites not listed in Figure 2, subsequent studies used 50% methanol to extract tissues (Figure 1A—bottom spectrum).

Perchloric acid extracts divalent cations, which broaden spectral peaks. The mantle (Figure 1B, top spectrum) and digestive gland/stomach demonstrated this broadened line width, while spectral quality was relatively sharp in extracts of adductor muscle tissue (Figure 1B, middle spectrum). The peaks of the C2 and C3 positions of the ^13^C serine isotopomers represented by the triplets centered at 57.3 ppm and 61.2 ppm, respectively, show a sharpening of the triplet and increased resolution in spectral quality from the methanol extract tissue. Treatment with Chelex™, a resin bead that chelates divalent metal ions, did not significantly increase resolution of the methanol tissue extract, suggesting that methanol sufficiently precipitates divalent cations, and that divalent cations were a likely cause of the broadening in this region. Because perchloric acid extract of adductor muscle tissue did not exhibit the same broadening of these resonances (Figure 1B, bottom spectrum) seen in the digestive gland/stomach, mantle, and gill tissue blocks (Figure 1B, top spectrum) suggests that the later tissues may contain higher concentrations of divalent metals. It is well documented that cadmium, iron, copper, and other cations are accumulated in oyster hepatopancreas and mantle [17,18,19,20,21].

### 2.2. Tissue Distribution of 2-^13^C/^15^N-Glycine and U-^13^C-Glucose in the Four Tissues and Treatments

The weight of the oyster and respective tissue blocks in the four treatments are presented in Table 1. The standard deviations for some of the tissue blocks, especially the gills, were very high, and we attribute this to using wet weights of tissues rather than reporting on a dry weight basis. The need to quench the metabolism rapidly did not permit the time to thoroughly dry the tissue. Dry weight reporting will be used in future studies to more accurately report of metabolite concentrations.

**Figure 3:**
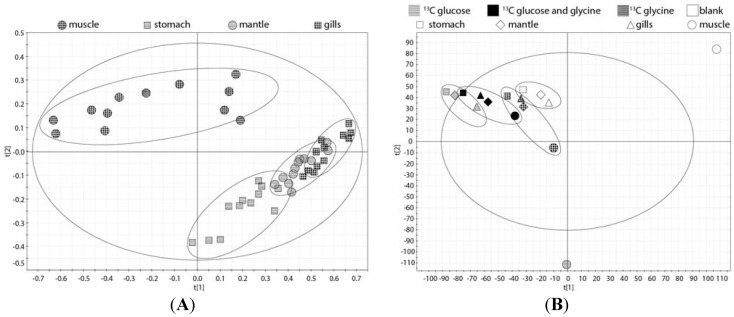
(**A**) The principal component analysis (PCA) of the 14 ^1^H spectra of the four oyster tissue blocks (mantle, gills, digestive gland/stomach, and muscle) treated with just seawater or seawater containing 2.7 mM 2-^13^C/^15^N-glycine, 5.5 mM U-^13^C-glucose, or 2.7 mM 2-^13^C/^15^N-glycine plus 5.5 mM U-^13^C-glucose; (**B**) The PCA plot of the ^13^C spectra of the four oyster tissue blocks from oyster exposed to the three treatments as in (**A**). Both 2.7 mM 2-^13^C/^15^N-glycine plus 5.5 mM U-^13^C-glucose is situated between the 2.7 mM 2-^13^C/^15^N-glycine (right) and 5.5 mM U-^13^C-glucose (left), indicating that the parent and metabolites are contributing to this separation.

In the present study, the molar ratios of the same 20 metabolites described in a previous ^1^H NMR metabolomic study of the Eastern oyster [5] were not significantly different between the three treatments. This result is corroborated by the principal component analysis (PCA) of the ^1^H NMR spectra of the four tissues displayed in Figure 3A showing little separation within each tissue. However, the PCA analysis of the ^1^H NMR spectra does reflect metabolic differences between the tissues especially the muscle compared to mantle, gills, and digestive gland/stomach (Figure 3A). In contrast, the PCA analysis of representative ^13^C NMR spectra of the four tissues displayed a clear difference in ^13^C metabolites between the three treatments (Figure 3B). This likely reflects the different spectral patterns of the parent ^13^C-labeled compound, 2-^13^C/^15^N-glycine and U-^13^C-glucose, and their metabolites.

**Table 1:** Weights of the four tissue blocks used in the 48 h uptake experiment using 2-^13^C/^15^N-glycine and U-^13^C-glucose, and the ^13^C fractional enrichment of alanine determined at the C3 position from the ^1^H NMR spectrum (see Experimental Section 3.2 and Section 3.6).

Treatment	N	Total *	Muscle	Gills	Stomach	Mantle
		Weight ± SD, g (%^13^C Ala ± SD)	Weight ± SD, g (%^13^C Ala ± SD)	Weight ± SD, g (%^13^C Ala ± SD)	Weight ± SD, g (%^13^C Ala ± SD)	Weight ± SD, g (%^13^C Ala ± SD)
Control	4	56.3 ± 13.2	0.43 ± 0.12	0.67 ± 0.10	1.26 ± 0.64	1.60 ± 0.59
		-	(ND)	(ND)	(ND)	(ND)
2-^13^C/^15^N-Glycine	3	53.5 ± 13.5	0.45 ± 0.18	1.03 ± 0.35	1.16 ± 0.11	1.39 ± 0.69
		-	(ND)	(ND)	(ND)	(ND)
U-^13^C-Glucose	4	55.5 ± 21.5	0.42 ± 0.23	0.63 ± 0.26	1.00 ± 0.65	0.89 ± 0.31
		-	(23% ± 3%)	(20% ± 3%)	(23% ± 10%)	(15% ± 2%)
2-^13^C/^15^N-Glycine + U-^13^C-Glucose	4	56 ± 10.8	0.48 ± 0.15	0.95 ± 0.12	1.23 ± 0.39	± 0.42
		-	(18% ± 2%)	(16% ± 2%)	(25% ± 12%)	(13% ± 2%)

* The average weight for each group of Eastern Oyster was made with water including shell and contained water, while the various organ blocks were weighed after dissection and prior to extraction. Abbreviations: N is the number of replicates per treatment; SD is standard deviation; ND is not detected.

### 2.3. Mass Balance of ^13^C-labeled Metabolites within Each of the Tissue Blocks

**Figure 4:**
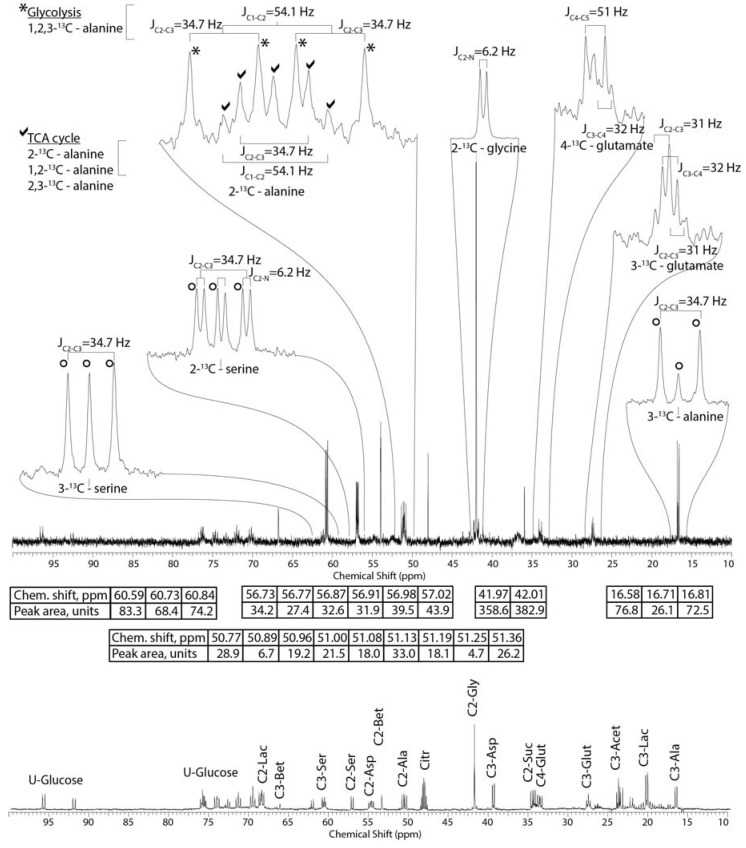
Representative ^13^C NMR spectra of methanol extract of mantle from oysters after 48 h of exposure to 2.7 mM 2-^13^C/^15^N-glycine and 5.5 mM U-^13^C-glucose in seawater. Inset spectral enlargements show the various coupling constants derived from the spectrum. Given in the tables below the spectrum are the respective chemical shifts and carbon-carbon coupling constants (J_cc_) of the various ^13^C isotopomers and associated peak areas. The ^13^C spectrum at the bottom of the figure identifies all ^13^C-labeled metabolites detected in this study, and is from the crushed oyster experiment discussed in the text.

Both glycine and glucose are metabolized in all four organ blocks in a similar manner, but glycogen is significantly ^13^C-labeled in the mantle and digestive gland/stomach for those treatments containing U-^13^C-glucose. Glycogen does not appear in the ^13^C spectrum shown in Figure 4 because it is precipitated during the methanol/water extraction process (see Section 2). When 2-^13^C/^15^N-glycine is applied, it is metabolized to serine. The supposition that serine is entirely formed from glycine is supported by the ^13^C-^15^N coupling constant (J_C-N_) of 6.3 Hz, which as expected is detected only in the resonance of the C2 and not C3 of serine. Also, the ratio of the serine isotopomers (*i.e*., 2,3-^13^C-serine, 2-^13^C-serine, and 3-^13^C-serine) is that expected if all of the glycine-derived serine was formed in the mitochondria [15]. Interestingly, although one of the metabolic pathways of betaine formation is trimethylation of glycine, we detect no ^13^C or ^15^N labeling of betaine, suggesting that at least under these metabolic conditions, betaine forms from other metabolic pathways or is assimilated directly in the diet. However, in the 2-^13^C/^15^N-glycine treatment, a minor amount of mitochondrial-derived serine forms alanine probably by entering glycolysis (spectrum not shown), and even less labeled aspartate and glutamate are formed via the Krebs cycle.

Based on our isotopomer analysis, all of the alanine in the 2-^13^C/^15^N-glycine + U-^13^C-glucose treatment is formed from glucose via glycolysis. From the various ^13^C-^1^H one bond-coupling constants, the primary alanine isotopomer is U-^13^C-alanine. We do not detect fractional enrichment of alanine obtained from the ^13^C-satelite peaks of the C3 position of alanine (Figure 1A) in the 2-^13^C-glycine treatments. In the two treatments containing U-^13^C-glucose (*i.e*., 2 mM U-^13^C-glucose and 2-^13^C-glycine plus U-^13^C-glucose), between 10%–25% of the alanine pool in the four tissue blocks (gills, mantle, muscle, and digestive gland/stomach) was labeled by the U-^13^C-glucose after three days of exposure (Table 1). Unlabeled glycogen, lipid or protein stores likely contributed, as the oysters were not fed during the 3 days of treatment and likely survived on endogenous energy stores. In fact, it is somewhat surprising that as much as a quarter of the alanine pool was labeled by exogenous glucose dissolve in the seawater. The discovery that glucose but not glycine contribute to alanine production is similar to previous enzyme studies quantifying alanine aminotransferase [22,23], but recent transcriptomic studies during environmental stress conditions have found up-regulating of alanine-glyoxylate transaminase. Future WoMBaP studies should include this dual ^13^C/^15^N labeling scheme of glycine to easily probe the AGT pathway in case this pathway becomes important during stress conditions.

**Figure 5:**
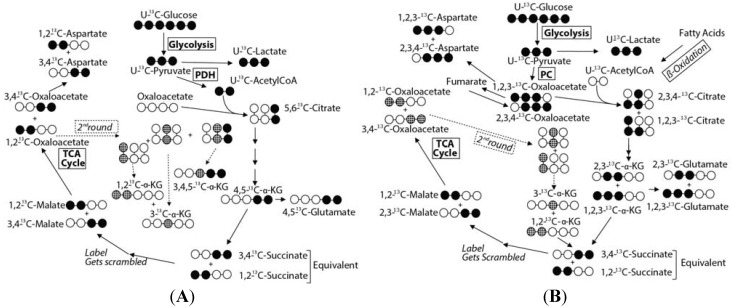
The metabolic scrambling of U-^13^C-glucose entering the Krebs cycle via pyruvate dehydrogenase (**A**) and pyruvate carboxylase (**B**) showing the pathway by which various isotopomers form. The ^13^C scrambling of labeled U-^13^C-pyruvate entering the Krebs cycle shows that the 1,2,3-^13^C-glutamate and 1,2,3-^13^C-aspartate isotopomers only form by entry into the Krebs cycle via pyruvate carboxylase, while 4,5-^13^C-glutamate and 3,4-^13^C-aspartate forms via pyruvate dehydrogenase.

The comparison of the levels of 1,2,3-^13^C-glutamate *versus* 4,5-^13^C-glutamate after administration of U-^13^C-glucose has been used as a relative measure of entry into the Krebs cycle via pyruvate carboxylase (PC) *versus* pyruvate dehydrogenase (PDH), respectively in rats, for over 25 years [24,25]. In our experiments, a significant amount of U-^13^C-pyruvate derived from U-^13^C-glucose enters the Krebs cycle labeling the C3 and C4 of glutamate (Figure 4), with entry via pyruvate dehydrogenase forming 4,5-^13^C-glutamate (Figure 5A) resulting in the C2-C3 coupling constant (J_C4-C5_ = 51 Hz) shown in Figure 4. A relatively large amount of 2,3-^13^C-glutamate forms via U-^13^C-pyruvate entering the Krebs cycle through the activity of pyruvate carboxylase (Figure 5B) [4]. We also detect a small amount of labeled glutamate at the C2 position (centered at 55.3 ppm), but due to insufficient signal-to-noise ratios, isotopomers are not quantified. However, the presence of a ^13^C label at the C2 position is detected through C2-C3 coupling constant (J_C2-C3_ = 31 Hz) shown in Figure 4. Also, the use of wet weights for organ blocks did not permit accurate calculation of concentrations and fractional enrichments. In future studies the ^13^C fractional enrichment of the ^13^C-labeled metabolites must be calculated.

### 2.4. Effect of Hypoxic Stress on the Oyster Mass Balance

**Figure 6:**
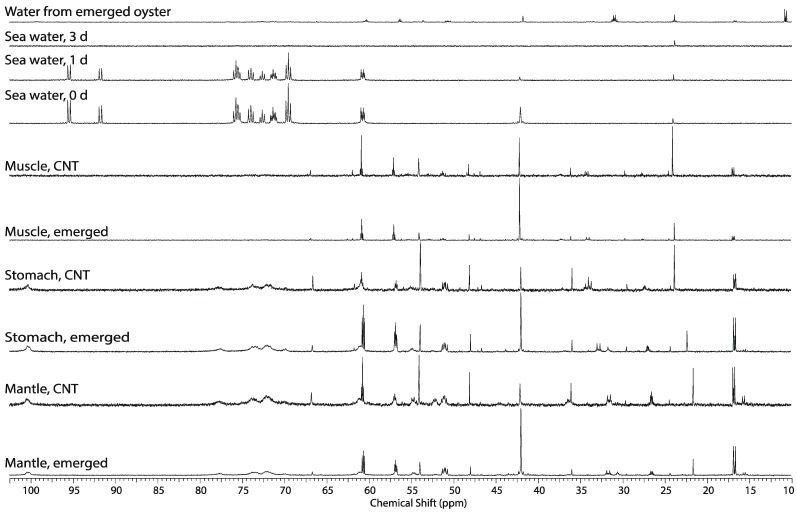
Representative ^13^C NMR spectra of the seawater at the start, 1 day and 3 days of exposure to 2.7 mM 2-^13^C/^15^N-glycine plus 5.5 mM U-^13^C-glucose under normal water and submerged conditions. After 24 h, slightly more glycine than glucose is consumed and bicarbonate at 163 ppm is labeled (not shown), likely formed from dissolved carbon dioxide, a catabolite of the Krebs cycle (third spectrum from top). By the third day, no glucose or glycine is detected in the seawater (second spectrum from top). Water from the shell of emerged or theoretically “hypoxic” oyster revealed that U-^13^C-glucose derived 1,2,3-^13^C-alanine, 1,2,3-^13^C-propionate (12.8, 33.4, 185 ppm), 1,2-^13^C-acetate (24 and 182.3 ppm) and 2-^13^C-glycine derived serine was secreted into this space (top spectrum); Representative ^13^C NMR spectra (10 ppm–105 ppm) of the perchloric acid extracts of oyster tissues (gills were included with the mantle for the emergence experiment) from control and oysters exposed to 12 h of emergence. Perchloric acid rather methanol extraction was used in order to extract the glycogen and demonstrate its tissue distribution. The chemical shift and concentration reference, 2-^13^C-acetate (singlet at 24 ppm) was added to the tissue extracts. Note that although 2-^13^C-acetate was added to the NMR samples as a concentration chemical shift reference the C2 acetate at 24 ppm in A and top spectrum is composed of a doublet representing 1,2-^13^C-,acetate.

As an example of WoMBaP application we investigate the oyster response to what we hypothesized to be hypoxia experienced at low tide when oysters are “emerged” and can be exposed to air. Alanine and serine isotopomers derived from U-^13^C-glucose and 2-^13^C-glycine, respectively, was detected in water from the shell of the emerged oysters (Figure 6A—top spectrum). However, the dominant metabolites were propionic acid and acetate derived from U-^13^C-glucose by propionic acid fermentation (Figure 6A—top spectrum).

In both emerged and submerged oysters the largest peaks in the ^13^C NMR spectra of the extracts of the three tissue blocks shown in Figure 6B are from U-^13^C-glucose derived alanine, the end-product of anaerobic glycolysis in oysters [26,27,28]*.* The most obvious difference between the spectra of the different tissue blocks of emerged oysters is that glycogen is present in the mantle (gills were included), and the digestive gland/stomach but not the adductor muscle (Figure 6B). In all tissues, either the natural ^13^C abundant osmolytes such as taurine and betaine decrease, or all of the ^13^C-labeled metabolites increase. In addition, based on the glutamate isotopomeric analysis, U-^13^C-pyruvate in these animals entered the Krebs cycle via pyruvate dehydrogenase and pyruvate carboxylase in all tissues. Other studies on oyster have shown the existence of pyruvate dehydrogenase, pyruvate carboxylase, malic enzyme, and PEP carboxykinase, and the effects of season and environmental stress [26,27]. Together these findings suggest that aerobic metabolism occurs throughout emergence. Because glutamate is produced in the Krebs cycle through aerobic metabolism, this suggests there was sufficient oxygen present in the emerged oyster. This may be occurring through micro-gaping behavior in air. The finding is in agreement with interpretations proposed for *in vivo* real-time ^31^P NMR studies of mussels [6]. Emerged conditions also cause concentrations of natural abundance betaine, an osmolyte, to decrease in all tissues (Figure 6B, compared control to emerged spectra).

Betaine, which is trimethylglycine, is not ^13^C-labeled and thus not derived from 2-^13^C-glycine. It should be noted that there were saturation effects evidenced by the C3 and C2 peak areas of serine wherein the dipolar relaxation of the C3 position has twice the protons and thereby a shorter T_1_ relaxation value than the C2 position [12]. The glycine cleavage system requires NAD^+^ and without oxygen NADH should accumulate as the electron transport chain is uncoupled and negative feedback slows the Krebs cycle. However, the ^13^C-labeled Krebs cycle anaplerotic product, isotopomers of glutamate, also increases in emerged oysters, suggesting that the Krebs cycle was not perturbed (Figure 6B). Glutamate isotopomers are the largest peaks in the seawater retained in the emerged oysters suggesting that the Krebs cycle is active during emergence and that these compounds were actively secreted into this intra-shell compartment. Surprisingly, the levels of glycogen in mantle and the digestive gland/stomach are not especially depleted with emergence. One would assume these compounds should be catabolized to glucose for consumption by glycolysis (Figure 6B, compare the four spectra second from the top).

**Figure 7:**
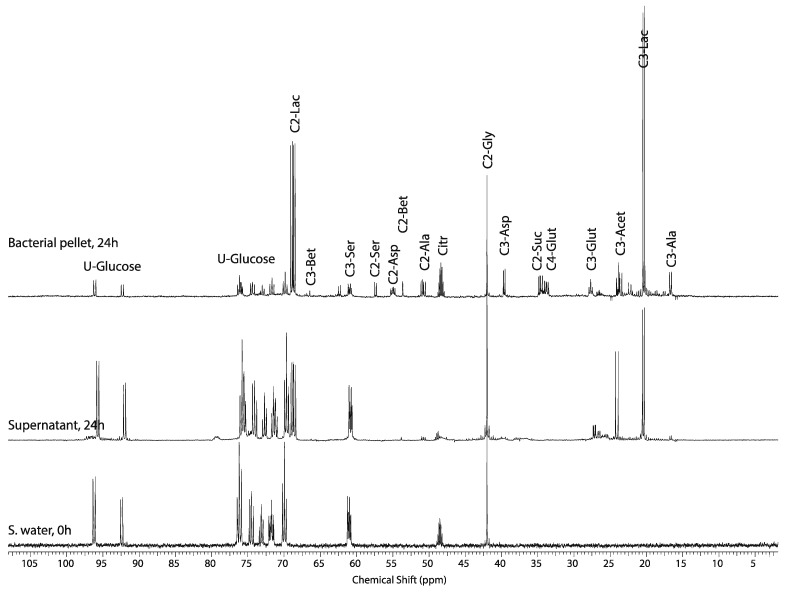
Representative ^13^C NMR spectra of the seawater from this incubation at the beginning of the experiment (bottom spectrum), and seawater supernatant (middle spectrum) and extracted pellet containing the oyster carcass (top spectrum) after centrifugation and 24 h, of incubation at room temperature. This study demonstrates the metabolism of 2-^13^C-glycine and U-^13^C-glucose by endemic bacteria creates significant levels of anaerobic glycolytic end-product, lactate (21 ppm), and additional metabolites (see text).

Earlier *in vivo* ^31^P NMR studies of marine mollusk showed that emergence and environmental stress causes a decrease of intracellular pH [6,7], and in vertebrates, lactate accumulates during anaerobic glycolysis decreasing intracellular pH, yet only alanine accumulates in mollusk. Metabolomic studies of the eastern oyster [5] have identified succinate and acetate as significant metabolites, and they increase in response to environmental stressors such as, temperature and ocean acidification [29], salinity [30], and hypoxia [14,31], affecting intracellular pH. To determine if bacteria endemic to the oyster or oyster symbionts are responsible for alanine production rather than the oyster**,** the oyster and its tissue was crushed in seawater containing 5.6 mM glucose with 2.7 mM glycine and allowed to metabolize for 24 h. The primary metabolite in the pellet (Figure 7, top spectrum), and seawater pellet (Figure 7, second spectrum from top) is U-^13^C-glucose derived lactate presumably from resident bacteria on or within the oyster. Comparing ^13^C NMR spectra from Figure 6B and Figure 7 (top spectrum), supports that the primary glycolytic end product of the oyster derived from either glucose or glycine is alanine not lactate. Although this is well established, it is interesting to highlight that bacteria were present in the exposure chamber exposing viable oysters, and the oyster either maintained low bacterial level by filtering the seawater and consuming them or inhibiting symbiont lactate production. This may be important in future environmental stress studies as hypercapnia has been shown to inhibit *vibrio* oyster infection [32], and simple analysis of lactate in the seawater could be a rapid easure of quality assurance in future stress studies. Also as expected, the indigenous bacteria ^13^C-label the various metabolites involved in the Krebs cycle producing citrate, succinate, aspartate, glutamate, acetate, and alanine from U-^13^C-glucose and serine from 2-^13^C-glycine (Figure 7—top spectrum).

### 2.5. Whole Body Mass Balance Phenotyping (WoMBaP) Summary Plot

**Figure 8:**
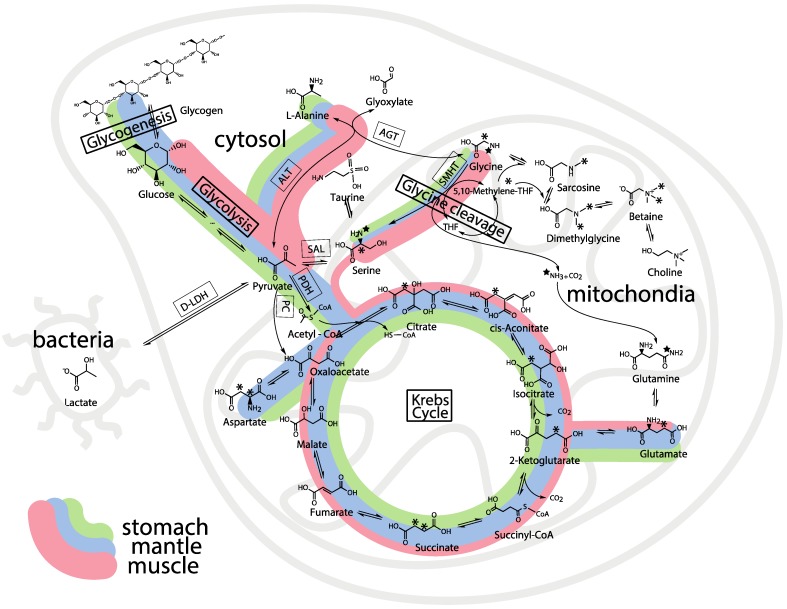
The whole body mass balance phenotyping (WoMBaP) summary plot of the oysters exposed for 3 days to 2.7 mM 2-^13^C/^15^N-glycine and 5.5 mM U-^13^C-glucose in seawater.

Figure 8 is a schematic overview of the tissue distribution and mass balance of 2-^13^C/^15^N-glycine and U-^13^C-glucose after 2 days of exposure. The metabolic pathways correspond to recent transcriptomic pathways in oyster exposed to similar conditions [13,14,16,31,33,34]. These color-coded schematic WoMBaP metabolic summary plots relate the amount of flux of glucose and glycine through the different metabolic pathways by the width of the line, while the color-coding represents the respective tissues and their relative amounts of whole body consumption by the different pathways of the glucose and glycine. This is a relatively easy format to relate entire quantitative fluxomic experiments and shifts in the mass balance of metabolites can be a measure of sub-lethal effects of environmental stress.

## 3. Experimental Section

### 3.1. Acquisition and Husbandry of Oysters

Unless otherwise stated, all chemicals used in this study were obtained from Sigma (St. Louis, MO, USA). Eastern Oysters (*Crassostrea virginica*) (50–70 g) were collected from Taylor Creek near Beaufort, NC, and fasted for one day in ﬁltered natural seawater prior to the experiments. A total of four oysters (n = 4) were used for each treatment of ^13^C labeled nutrients (as: blank, ^13^C glucose 5.5 mM, ^13^C glycine 2.7 mM, and both ^13^C glucose plus ^13^C glycine).

### 3.2. WoMBaP Experimental Procedure

A total of sixteen oysters were analyzed by NMR spectroscopy, one gave very little signal for muscle and gills and was exclude from analysis (Table 1). Experiments were conducted in October 2010. Seawater with salinity 29–32 ppt was obtained from Bogue Sound at the Center for Marine Sciences and Technology (CMAST), ﬁltered through a 0.2 µm ﬁlter, and then used immediately in the experiments. After fasting for a day, oysters were transferred to a beaker containing 200 mL of water and the appropriate ^13^C labeled nutrients (5.5 mM ^13^C glucose, 2.7 mM ^13^C glycine). Aeration was provided with a 1/4 inch diameter Tygon™ (Aqueon Products, Franklin, WI, USA) tube inserted into each beaker bubbling room air lightly to avoid startling the oyster into closing. Oysters were maintained at room temperature for 48 h prior to dissection.

### 3.3. Dissection and Extraction of Oyster Tissue Blocks

Four tissue blocks ((1) muscle; (2) GI with digestive gland; (3) mantle with heart; (4) gills) were harvested within 30 s of quickly opening the shell using an oyster knife. The harvested blocks were immediately frozen in liquid nitrogen. Each organ block was pulverized individually using a stainless steel mortar and pestle with constant addition of liquid nitrogen. The crushed organ block was then transferred to a 50 cc polyethylene disposable centrifuge tube and weighed. For perchloric acid extraction, 8% perchloric acid was added 2-to-1 *v:w* (volume:weight). Methanol extractions were performed by adding 50% methanol to the sample suspended in water to achieve a 4-to-1 *v:w* ratio. This mixture was vortexed for 1 min, before being refrigerated overnight (4 °C). After centrifugation at 4,500 g for 5 min, the supernatant was collected, and the pH adjusted with series of potassium hydroxide concentrations to precipitate perchlorate salts and achieve a slightly alkaline pH between pH 7–7.4. The supernatant was then centrifuged again at 4,500 g for 5 min to settle any precipitate. The clear supernatant was then lyophilized in a 50 cc polyethylene disposable tube and stored in a plastic cryovial at −80 °C until spectroscopy was performed. Even though drying the tissue prior to extraction and operating with dry weights giving more reliable concentration measurements, some of the compounds can oxidize and evaporate during the lyophilization. So in this study we’ve quenched metabolism and extracted the tissues immediately after harvesting and weighting, which resulted in using wet tissue weights and molar rations.

### 3.4. Emergence and Crushed Oyster Experimental Procedure

To investigate whether lactate accumulates with emergence and if it forms from endemic bacteria, two experiments were performed: (1) two oysters for the emergence study and (2) one oyster for the indemic bacteria study. For the emergence study, the oysters were exposed for 48 h to 2.7 mM 2-^13^C/^15^N-glycine plus 5.5 mM U-^13^C-glucose under normal conditions at room temperature (~23 °C) in the dosing chamber. The control oyster remained in the perfusion system, while the “hypoxic” oyster was removed from water, and kept emerged for 12 h at room temperature simulating a “double low tide”. After the 12 h the oysters were dissected and extracted. In order to expedite metabolic quenching the gills were not dissected and included with the mantle, so only three tissue blocks ((1) muscle; (2) GI with digestive gland; (3) mantle with heart and gills) were analysed. For the endemic bacteria oyster experiment, the oyster tissue was crushed to a fine suspension and filtered through 200 μm nylon mesh and the filtrate was incubated with 2.7 mM 2-^13^C/^15^N-glycine plus 5.5 mM U-^13^C-glucose in 50 mL filtered seawater in a beaker without bubbling. After 12 h, the suspension was centrifuged at 4,500 g for 5 min and the pellet was extracted with perchloric acid, while the supernatant lyophilized directly.

### 3.5. ^1^H and ^13^C NMR Spectroscopy of Extracted Tissue Blocks

Lyophilized powder from the perchloric acid extraction for each of the organ blocks for each oyster was dissolved in 0.7 mL deuterium oxide with 0.2% TSP and transferred to a 5 mm NMR tube. The ^1^H NMR spectra were acquired at 25 °C using a 14.1 T Varian Inova spectrometer equipped with a 5 mm HCN NMR probe with a one-pulse sequence using a 90° ﬂip angle, with a 1.5 s presaturation pulse on residual water, a 2.5 s acquisition time and 1.5 s relaxation time resulting in a 4 s repetition time. The sweep width was 6,000 Hz and acquired with 15,000 complex points, and 128 transients. The ^13^C NMR spectra were acquired at 25 °C using a 14.1 T Varian Inova spectrometer equipped with a 5 mm broadband NMR probe with a one-pulse sequence with WALTZ decoupling during acquisition, a 2 s acquisition time and 2 s relaxation time resulting in 4 s repetition time. The sweep width was 32,000 Hz and acquired with 64,000 complex points. For the WoMBaP studies there were 512 transients and for the emergence studies there were 1,024 transients.

### 3.6. Analysis of ^1^H and ^13^C NMR Spectra and Generation of WoMBaP Summary Plots

Data were analyzed using an ACD Labs 9.0 1D NMR Processor (ACD Labs). ^1^H spectra were zero-ﬁlled to 32,000 points, and line broadened using a 0.3 Hz exponential Gaussian function. Metabolites were identified in the ^1^H NMR spectra based on previous studies of the eastern oyster metabolome [5]. Though we attempted to calculate concentrations based on the ^1^H spectrum by comparing areas under peaks to TSP peaks as previously described [35], unfortunately our use of wet weights for the tissue blocks contributed excessive variability, most likely because of the presence of extra sea water, particularly for the gills. Therefore, rather than concentrations, molar ratios were established by dividing the area under a peak by the sum of all peak areas in the spectrum, excluding the water peak. NMR determination of molar ratios is a conventional and validated method for metabolic profiling [36,37]. The ^13^C fractional enrichments were obtained from the ^13^C satellite peaks in the ^1^H NMR spectra [38]. For the Principal Component Analysis, spectra were binned into 0.005 ppm buckets, using whole spectrum area as a reference. TSP (<0.5 ppm), water (4.7–4.9 ppm) and >9.5 ppm regions were excluded. Data were moved to the SIMCA P+ software (Umetrics, Umea, Sweden) where Principal Component Analysis on X-block was performed. First and second component were used to make the score scatter plot. The WoMBaP summary plots were generated by multiplying the weight of each tissue by the ^13^C fractional concentration of 2-^13^C-glycine and U-^13^C-glucose, and expressed as the thickness of the colored line. In the present study, the molar ratios from the ^1^H and ^13^C spectra were used to avoid the high variability encountered with the wet weights (Table 1).

## 4. Conclusions

The tissue distribution and disposition of 2-^13^C-glycine and U-^13^C-glucose in *C. virginica* was determined. Isopopmeric analysis revealed that glucose was primarily consumed in glycolysis and the Krebs cycle forming alanine, and glutamate and aspartate, respectively. As expected, alanine is the end product of glycolysis in oysters rather than lactate, while lactate is the end product for many bacteria found in seawater. Alanine was produced via alanine transaminase and not alanine-glyoxylate transaminase. Glycine was converted primarily to serine via the glycine cycle and serine was entirely formed in the mitochondria. These initial isotopomer studies form the basis of a new fluxomic phenotyping method, WoMBaP, which has potential for elucidating sub-lethal environmental stressors. In future, studies using a dry weight basis for concentration calculations, and the use of a fast 2D NMR technique rather than 1D ^13^C and ^1^H NMR spectroscopy, should be considered.

## References

[1] Smart R.C., Hodgeson E. (2008). Molecular and Biochemical Toxicology.

[2] Klaassen C.D. (2007). Casarett & Doull’s Toxicology: The Basic Science of Poisons.

[3] Lee H., Tikunov A., Stoskopf M.K., Macdonald J.M. (2010). Applications of chemical shift imaging to marine sciences. Mar. Drugs.

[4] Tikunov A.P., Winnike J.H., Tech K., Jeffries R.E., Semelka C.T., Martin J., McClelland R., Graves L.M., Macdonald J.M. (2013). Fluxomics by NMR spectroscopy from cells to organisms focusing on liver. Curr. Metabolomics.

[5] Tikunov A., Johnson C.B., Lee H., Stoskopf M.K., Macdonald J.M. (2010). Metabolomic investigations of American oysters Using ^1^H-NMR spectroscopy. Mar. Drugs.

[6] Fan T.W., Higashi R.M., Macdonald J.M. (1991). Emergence and recovery response of phosphate metabolites and intracellular pH in intact *Mytilus edulis* as examined *in situ* by *in vivo*
^31^P-NMR.**. Biochim. Biophys. Acta (BBA)—Mol. Cell Res..

[7] Higashi R.M., Fan T.W.-M., Macdonald J.M. (1989). Monitoring of metabolic responses of intact Haliotis (abalones) under salinity stress by ^31^P surface probe localized NMR. J. Exp. Zool..

[8] Hines A., Oladiran G.S., Bignell J.P., Stentiford G.D., Viant M.R. (2007). Direct sampling of organisms from the field and knowledge of their phenotype: Key recommendations for environmental metabolomics. Environ. Sci. Technol..

[9] Jones O.A.H., Spurgeon D.J., Svendsen C., Griffin J.L. (2008). A metabolomics based approach to assessing the toxicity of the polyaromatic hydrocarbon pyrene to the earthworm Lumbricus rubellus. Chemosphere.

[10] Tuffnail W., Mills G.A., Cary P., Greenwood R. (2009). An environmental ^1^H NMR metabolomic study of the exposure of the marine mussel *Mytilus edulis* to atrazine, lindane, hypoxia and starvation. Metabolomics.

[11] Viant M.R., Bundy J.G., Pincetich C.A., de Ropp J.S., Tjeerdema R.S. (2005). NMR-derived developmental metabolic trajectories: An approach for visualizing the toxic actions of trichloroethylene during embryogenesis. Metabolomics.

[12] Macdonald J.M., Schmidlin O., James T.L. (2002). *In vivo* monitoring of hepatic glutathione in anesthetized rats by ^13^C NMR. Magn. Reson. Med..

[13] Jo P.G., Choi Y.K., Choi C.Y. (2008). Cloning and mRNA expression of antioxidant enzymes in the Pacific oyster, *Crassostrea gigas* in response to cadmium exposure. Comp. Biochem. Physiol. Part C.

[14] Ivanina A.V., Sokolov E.P., Sokolova I.M. (2010). Effects of cadmium on anaerobic energy metabolism and mRNA expression during air exposure and recovery of an intertidal mollusk *Crassostrea virginica*. Aquat. Toxicol..

[15] Johnson C.B., Tikunov A.P., Lee H., Wolak J.E., Pediaditakis P., Romney D., Holmuhamedov E., Gamcsik M.P., Macdonald J.M. (2012). ^13^C MRS detection of changes in serine isotopomers reflects changes in mitochondrial redox status. Magn. Reson. Med..

[16] Meng J., Zhu Q., Zhan L., Li C., Li L., She Z., Huang B., Zhang G. (2013). Genome and transcriptome analyses provide insight into the euryhaline adaptation mechanism of *Crassostrea gigas*. PLoS One.

[17] Cunningham P.A., Tripp M.R. (1975). Accumulation, tissue distribution and elimination of ^203^HgCl_2_ and CH_3_
^203^HgCl in the tissues of the American oyster *Crassostrea virginica*. Mar. Biol..

[18] Denton G.R.W., Burdon-Jones C. (1981). Influence of temperature and salinity on the uptake, distribution and depuration of mercury, cadmium and lead by the black-lip oyster *Saccostrea echinata*. Mar. Biol..

[19] Martincié D., Nürnberg N.W., Stoeppler M., Branica M. (1984). Bioaccumulation of heavy metals by bivalves from Lim Fjord (North Adriatic Sea). Mar. Biol..

[20] Bryan G.W., Langston W.J. (1992). Bioavailability, accumulation and effects of heavy metals in sediments with special reference to United Kingdom estuaries: A review. Environ. Pollut..

[21] Kurochkin I.O., Ivanina A.V., Eilers S., Downs C.A., May L.A., Sokolova I.M. (2009). Cadmium affects metabolic responses to prolonged anoxia and reoxygenation in eastern oysters (*Crassostrea virginica*). Am. J. Physiol. Regul. Integr. Comp. Physiol..

[22] Paynter K.T., Karam G.A., Ellis L.L., Bishop S.H. (1985). Subcellular distribution of aminotransferases, and pyruvate branch point enzymes in gill tissue from four bivalves. Comp. Biochem. Physiol. Part B: Comp. Biochem..

[23] Donald K.M., Hawkins A.J.S., Smerdon G.R. (2001). Transcript analysis of the genes encoding aminopeptidase N and alanine aminotransferase, two enzymes involved in protein turnover, in the pacific oyster, *Crassotrea gigas*. Comp. Biochem. Physiol..

[24] Katz J., Lee W.-N., Wals P.A., Bergner E.A. (1989). Studies of glycogen synthesis and the Krebs cycle by mass isotopomer analysis with [U-^13^C]Glucose in rats. J. Biol. Chem..

[25] Uehara T., Kosyk O., Jeannot E., Bradford B.U., Tech K., Macdonald J.M., Boorman G.A., Chatterjee S., Mason R.P., Melnyk S.B. (2013). Acetaminophen-induced acute liver injury in HCV transgenic mice. Toxicol. Appl. Pharmacol..

[26] Greenway S.C., Storey K.B. (2000). Seasonal change and prolonged anoxia affect the kinetic properties of phosphofructokinase and pyruvate kinase in oysters. J. Comp. Physiol..

[27] Le Moulla G., Bacca H., Huvet A., Moal J., Pouvreau S., van Wormhoudt A. (2007). Transcriptional regulation of pyruvate kinase and phosphoenolpyruvate carboxykinase in the adductor muscle of the oyster *Crassostrea gigas* during prolonged hypoxia. J. Exp. Zool..

[28] Hammen C.S. (1969). Metabolism of the oyster, *Crassostrea virginica*. Am. Zool..

[29] Lannig G., Eilers S., Pörtner H.O., Sokolova I.M., Bock C. (2010). Impact of ocean acidification on energy metabolism of oyster, *Crassostrea gigas*-changes in metabolic pathways and thermal response. Mar. Drugs.

[30] Dickinson G.H., Ivanina A.V., Matoo O.B., Pörtner H.O., Lannig G., Bock C., Beniash E., Sokolova I.M. (2012). Interactive effects of salinity and elevated CO2 levels on juvenile eastern oysters, *Crassostrea virginica*. J. Exp. Biol..

[31] Sussarellu R., Fabioux C., le Moullac G., Fleury E., Moraga D. (2010). Transcriptomic response of the Pacific oyster *Crassostrea gigas* to hypoxia. Mar. Genomics.

[32] Macey B.M., Achilihu I.O., Burnett K.G., Burnett L.E. (2008). Effects of hypercapnic hypoxia on inactivation and elimination of *Vibrio campbellii* in the Eastern oyster, *Crassostrea virginica*. Appl. Environ. Microbiol..

[33] Chapman R.W., Mancia A., Beal M., Veloso A., Rathburn C., Blair A., Holland A.F., Warr G.W., Didinato G., Sokolova I.M. (2011). The transcriptomic responses of the eastern oyster, *Crassostrea virginica*, to environmental conditions. Mol. Ecol..

[34] David E., Tanguy A., Pichavant K., Moraga D. (2005). Response of the Pacific oyster *Crassostrea gigas* to hypoxia exposure under experimental conditions. FEBS J..

[35] Dewar B.J., Keshari K., Jeffries R., Dzeja P., Graves L.M., Macdonald J.M. (2010). Metabolic assessment of a novel chronic myelogenous leukemic cell line and an imatinib resistant subline by ^1^H NMR spectroscopy. Metabolomics.

[36] Fan T.W.M., Colmer T.D., Lane A.N., Higashi R.M. (1993). Determination of metabolites by *1*H NMR and GC: Analysis for organic osmolytes in crude tissue extracts. Anal. Biochem..

[37] Fan T.W.M., Higashi R.M., Lane A.N., Jardetzky O. (1986). Combined use of ^1^H-NMR and GC-MS for metabolite monitoring and *in vivo*
^1^H-NMR assignments. Biochim. Biophys. Acta.

[38] Szyperski T. (1998). ^13^C-NMR, MS and metabolic flux balancing in biotechnology research. Q. Rev. Biophys..

